# Development and Analysis of a Hydroxyapatite Supplemented Calcium Silicate Cement for Endodontic Treatment

**DOI:** 10.3390/ma15031176

**Published:** 2022-02-03

**Authors:** David Yong, Joanne Jung Eun Choi, Peter Cathro, Paul R. Cooper, George Dias, Jeffrey Huang, Jithendra Ratnayake

**Affiliations:** 1 Faculty of Dentistry, Sir John Walsh Research Institute, University of Otago, P.O. Box 56, Dunedin 9054, New Zealand; david.yong@postgrad.otago.ac.nz (D.Y.); joanne.choi@otago.ac.nz (J.J.E.C.); peter.cathro@otago.ac.nz (P.C.); p.cooper@otago.ac.nz (P.R.C.); 2 Department of Anatomy, School of Biomedical Sciences, University of Otago, P.O. Box 56, Dunedin 9054, New Zealand; george.dias@otago.ac.nz (G.D.); huaje474@student.otago.ac.nz (J.H.)

**Keywords:** bovine derived hydroxyapatite, calcium silicate cement, compressive strength, radiopacity, biocompatibility

## Abstract

**Aim:** To develop an endodontic cement using bovine bone-derived hydroxyapatite (BHA), Portland cement (PC), and a radiopacifier. **Methods:** BHA was manufactured from waste bovine bone and milled to form a powder. The cements were developed by the addition of BHA (10%/20%/30%/40% wt), 35% wt, zirconium oxide (radiopacifier) to Portland cement (PC). A 10% nanohydroxyapatite (NHA) cement containing PC and a radiopacifier, and a cement containing PC (PC65) and a radiopacifier were also manufactured as controls. The cements were characterised to evaluate their compressive strength, setting time, radiopacity, solubility, and pH. The biocompatibility was assessed using Saos-2 cells where ProRoot MTA acted as the control. Compressive strength, solubility and pH were evaluated over a 4-week curing period. **Results:** The compressive strength (CS) of all cements increased with the extended curing times, with a significant CS increase in all groups from day 1 to day 28. The BHA 10% exhibited significantly higher CS compared with the other cements at all time points investigated. The BHA 10% and 20% groups exhibited significantly longer setting times than BHA 30%, 40% and PC65. The addition of ZrO_2_ in concentrations above 20% wt and Ta_2_O_5_ at 30% wt resulted in a radiopacity equal to, or exceeding that of, ProRoot MTA. The experimental cements exhibited relatively low cytotoxicity, solubility and an alkaline pH. **Conclusions:** The addition of 10% and 20% BHA to an experimental PC-based cement containing 35% ZrO_2_ improved the material’s mechanical strength while enabling similar radiopacity and biocompatibility to ProRoot MTA. Although BHA is a cost-effective, biomimetic additive that can improve the properties of calcium silicate endodontic cements, further studies are now warranted to determine its clinical potential.

## 1. Introduction

Mineral trioxide aggregate (MTA) is a calcium silicate cement (CSC) with wide-ranging endodontic applications. Following the important stages of chemical disinfection using chemicals such as sodium hypochlorite, it has been used for procedures such as perforation repair, revascularisation and use as an apical plug for the management of large and open-apices (apexification) [1,2,3,4], and is a root-end filling material [5,6]. It is considered the gold standard material for vital pulp therapy [7,8]. ProRoot MTA consists of an approximate 4:1 ratio of modified Portland cement (PC) to the radiopacifier, bismuth oxide (BIO) (21.8%). Pure PC, also known as clinker, is comprised of tricalcium aluminate, tetracalcium aluminofluorate and tricalcium silicate, dicalcium silicate, and is often combined with calcium sulphate and calcium carbonate [9]. The chemical similarity and low cost of PC make it an ideal alternative material to MTA for experimental research [10]. When PC or MTA are combined with water, calcium silicate hydrate (C-S-H) and calcium hydroxide (CH) are produced [11]. C-S-H provides the set material with physical strength, while calcium hydroxide dissociates into hydroxyl (OH) ions with antimicrobial properties and calcium ions which reportedly induce progenitor cell differentiation important for the repair of dental hard tissues [12,13]. Despite MTA’s clinical properties, the material’s high cost, difficult handling properties and tendency to discolour teeth has led to studies aimed at developing modified formulations [14]. Rheology modifiers, i.e., polycarboxylate ethers, have been added to improve the workability and handling properties of CSCs [15]. Zirconium oxide has been proposed as an alternative to the radiopacifier BIO which has been shown to stain dentine [16]. Calcium phosphate materials have also been added to CSCs to improve biomineralisation and apatite formation [17].

Calcium phosphate cements (CPCs) are used primarily in orthopaedic applications and, in contrast to CSCs, they have excellent biocompatibility and bioresorbability. However, CPCs exhibit poor mechanical strength, making them unsuitable for load-bearing applications [18]. Calcium phosphate monobasic (CPM) has been incorporated into CSCs as it reacts with the CH released from CSCs in an acid-base reaction to form carbonated HA in physiological solutions [17]. The formation of apatite purportedly induces osteogenesis, cementogenesis, cell differentiation and tissue repair, and improves the sealing ability [17,19]. In low concentrations (10–20%), CPM has also been shown to improve the compressive strength of some CSCs [20]. The consumption of free CH results in a concomitant drop in the materials setting pH and calcium ion release [21]. A reduction in the release of these ions can result in impaired antimicrobial and biological properties [22].

Hydroxyapatite (HA) is a natural component of enamel, dentine and bone, and is a biomimetic and more chemically stable alternative to CPM [23]. The addition of HA to CSCs may be an alternative way to increase apatite content without reducing the release of CH. HA on its own has shown promise as an alternative direct pulp capping material in several animal [24,25,26] and human studies [27,28]. HA has also been used as a component in bone grafting and restorative materials, as well as root canal sealers and dentifrices [29]. Thus far, the effect of HA addition on CSCs has not been studied extensively. However, notably, in a recent study, the addition of 5–15% HA in the form of nanohydroxyapatite (NHA) significantly increased the compressive strength of an experimental CSC [30]. In contrast, Guerriro-Tanomaru and Vazquez-Garcia reported that the addition of 10–20% NHA reduced the compressive strength and increased the solubility of a CSC-based material [31]. These conflicting results suggest that further research is required to better understand the effect that HA addition has on the physical and chemical properties of CSCs. 

Our previously published work reported the development of a low cost process for extracting hydroxyapatite from waste bovine bone (BHA), which exhibits a chemical composition similar to that derived from human bone [32]. To our knowledge, no previous research has been conducted investigating the physicochemical effects of BHA addition to a CSC. An experimental radiopacified endodontic cement was developed for this purpose using BHA, PC, a non-staining radiopacifier (ZO) and a plasticising agent (PCE) to improve material handling. The aim of this research was to investigate whether the addition of BHA enhances the mechanical, physicochemical and biocompatibility properties of an experimental radiopacified PC.

## 2. Materials and Methods

The prepared BHA cements were chemically characterised and analysed for their physical, chemical and radiographic properties (compression strength, setting time, radiopacity, solubility and pH) using methods adapted from Torabinejad, Hong [33]. The in-vitro biocompatibility of BHA cements was compared with ProRoot MTA using the Saos-2 osteoblast-like cell line.

### 2.1. Preparation of BHA Powders

BHA powder was produced using a method adapted from Ratnayake et al. (2017) [32]. Briefly, the bovine femoral condyle was dissected into 3–5 cm cubes, then pressure cooked for 2 h, followed by immersion in 0.1 M NaOH solution for 12 h at 70 °C. This process was repeated a second time to remove fat and connective tissue. The cubes were thoroughly cleaned using distilled water, microwaved for 5 mins (2.45 GHz, 1100 W, Samsung, Auckland, New Zealand) and sintered at 800 °C for 8 h in a muffle furnace (Daihan FH-14 furnace) at a heating rate of 20 °C per minute. Dried cubes were grounded using an agate mortar and pestle and a ball milled to produce a fine powder. The resulting powder was sieved using a 150 µm stainless steel sieve (Labsupply, Auckland, New Zealand) [34,35].

### 2.2. Preparation of Cements

Six cement combinations were analysed, consisting of a radiopacifier, BHA or synthetic nanohydroxyapatite (NHA) (Acros Organics, Auckland, New Zealand) and Portland cement (PC) (EverSure GP, Golden Bay Cement, Auckland, New Zealand) composed of 91% clinker, 4% gypsum (CaSO_4_) and 5% limestone (CaCO_3_). Zirconium dioxide (ZrO_2_) (Sigma Aldrich, Auckland, New Zealand) was used in all studies except for the radiopacity analysis where ProRoot MTA and a range of ratios of ZrO_2_ or tantalum pentoxide (Ta_2_O_5_) (Sigma Aldrich, Auckland, New Zealand) were also compared. The HA: PC: Zr ratios and powder/liquid ratios used are presented in Table 1. Pilot studies were conducted to adjust the powder/liquid ratios to produce a putty-like consistency for ease of manipulation. The liquid component consisted of 98% distilled water and 2% PC-707 superplasticiser (Stratmore Construction, Auckland, New Zealand).

#### 2.2.1. Chemical Characterisation

##### Inductively Coupled Plasma Mass Spectrometry (ICP-MS)

The elemental composition of BHA 10% was analysed using ICP-MS (Agilent 7500ce quadrupole, Tokyo, Japan) after immersion in distilled water for 28 days at 37 °C. One hundred milligrams of sample was crushed and digested at 104 °C for 1 h in quartz using nitric acid (Sigma Aldrich, Auckland, New Zealand). Following digestion, the samples were cooled to room temperature and diluted prior to analysis using an Agilent 7500ce quadrupole ICP-MS [34].

##### Fourier Transform Infrared Spectroscopy (FT-IR)

BHA, NHA, PC, ZrO_2_ powders, and BHA 10% and 20% cements were investigated under Attenuated Total Reflectance-FTIR spectroscopy to characterise their chemical composition. A Spectrometer (ATR-FTIR Alpha II, Bruker, Berlin, Germany) was used in the region of 400–4000 cm^−1^ with a resolution of 4 cm^−1^ and 64 scans for each spectrum. The ATR area had a 2 mm diameter and the IR radiation penetration was approximately 3–5 μm. Moderate pressure (5 psi) was applied to the specimens during measurements, ensuring adequate contact with the ATR crystal to reduce background noise and attain high quality spectra.

##### Scanning Electron Microscopy (SEM) and Energy Dispersive X-Ray Spectroscopy (EDX) Analysis

Scanning electron microscope (SEM) photomicrographs were captured at 150 × magnification for BHA (10%, 20%, 30%, 40%), NHA, MTA and PC65 cements after initial setting and at 21 days immersion in distilled water. Specimens were prepared for observation under SEM by mounting them on aluminium stubs, using double-sided carbon tape. They were coated with ~10 nm of carbon in a Peltier-cooled high-resolution sputter coater (Emitech K575X; EM Technologies Ltd., Tokyo, Japan) fitted with a carbon coater (Emitech 250X; EM Technologies Ltd.). Analysis was conducted by field emission SEM (Zeiss Sigma VP FEG SEM; Zeiss, Tokyo, Japan). SEM-EDS analysis was performed on BHA, NHA, PC and ZrO_2_ powders and set MTA cement, to evaluate their chemical compositions.

#### 2.2.2. Mechanical and Physicochemical Properties

##### Compressive Strength

Compressive strength testing was performed for each of the six experimental types of cement (BHA10/20/30/40/NHA/PC65) using the ISO 9917-1 protocol [36]. The powder and liquid components were combined according to established ratios (see Table 1) and packed into a lubricated polycarbonate split mould (4 mm diameter × 6 mm) using a spatula and amalgam plugger to a slight excess. The moulds were then compressed between two acrylic plates using a vice, then removed from the vice and smoothed flat using a spatula and a moistened cotton gauze. After 24 h, the cylinders for each group (*n* = 30) were removed from moulds, inspected and immersed in distilled water at 37 °C for 1, 7 or 28 days. Compression testing to failure was carried out in an Instron universal testing machine (Instron 3369; Norwood, MA, USA), using a 5 kN (±2 N) load cell at a crosshead speed of 1 mm/min. The mean value from 10 compression tests per test group was used to calculate the compressive strength (MPa) of each material.

##### Radiopacity

Radiographic density was determined in equivalent aluminium thickness in millimetres (mm), and performed for each of the six experimental cements: ProRoot MTA, plain Portland cement (PC) and PC + 15%, 20%, 25%, 30%, 35%, 40% of either Ta_2_O_5_ or ZrO_2_. ISO 6876 was used to assess radiopacity in each sample [37]. Three samples per cement were fabricated in 8 mm × 1 mm discs using a method adapted from Borges, Pedro to fit 8 samples on a size 2 periapical photostimulable storage phosphor (PSP) plate (Dentsply Sirona) [38]. Samples were exposed adjacent to a 97.9% pure aluminium stepwedge (Magraf Dental) with nine 1 mm step increments at a film to focus distance of 30 cm from a heliodent plus (Dentsply Sirona) X-ray source (70 kVp, 7 mA, 0.10 s), and processed with a Xios PSP Scanner (Dentsply Sirona, York, UK) using Sidexis imaging software prior to exporting as a JPEG file. ImageJ software version 1.8 (National Institutes of Health, Bethesda, MD, USA) was used to generate a calibration curve, converting the radiographic density values to an aluminium step wedge equivalent thickness (mm Al). The radiographic density of each cement was reported as the average value obtained from two discs exposed twice.

##### Setting Time

The ISO 6876 (2012) standard using a conditioned gypsum mould was used to test the setting time for all the cement types (Table 1). Freshly mixed gypsum was placed in a petri dish, and a plastic disc (10 mm diameter, 1 mm height) was placed in the unset gypsum. The disc was removed after the setting was complete, leaving an indented cavity 10 mm diameter by 1 mm deep. The gypsum mould was stored for 24 h prior to testing at 37 °C in 100% humidity, then soaked in water for 1 h prior to testing, and excess moisture was removed with a paper towel. The cement was then mixed and placed into the cavity and dried, and Vicat apparatus (Humboldt Mfg, Elgin, IL, USA) with a flat-ended 1.0 mm and 300 mg weight was used to indent, initially every 5 min, and then every minute after signs of setting were observed. 

In between indentations, the moulds were placed on a temperature-controlled hotplate at 37 °C and covered with damp gauze to prevent dehydration. The test was repeated three times per cement, and setting time was calculated as an average of the time taken from end of mixing to the time when the needle failed to make a complete circular indentation in the material.

##### Solubility and pH 

A method developed by Carvalho-Junior and Correr-Sobrinho, and validated for use with a calcium silicate cement (Biodentine) was applied [39,40]. For each sample, 6 discs (7.75 mm diameter × 1.5 mm) were fabricated in a mould and suspended vertically in 7.5 mL of distilled water in a sealed glass vial. 

Cements were mixed and placed in moulds with a slight excess, then compressed between glass plates and cured at 37 °C, in 100% humidity for 24 h. Discs were weighed using a precision balance (Voyager Pro Balance, Ohaus, NJ, USA) prior to immersion vertically in deionised water, and re-weighed at intervals of 7, 14, 21 and 28 days. Samples were dried on filter paper in oven at a temperature of 50 °C for 6 h prior to weighing. At each time point the pH was tested using a temperature calibrated pH probe (Milwaukee MW 101, Milwaukee, WI, USA). 

#### 2.2.3. In Vitro Biocompatibility Testing

The biocompatibility of the ProRoot MTA and BHA (10, 20, 30, 40%) cements were assessed using Saos-2 (ATCC HTB-85, USA) human osteoblast-like cells in conjunction with the LIVE/DEAD**^®^** cell viability/cytotoxicity assay (Invitrogen Life Technologies, Carlsbad, CA, USA) and the MTS assay (CellTiter 96**^®^** Aqueous One Solution, Promega, WI, USA).

##### Cement Disc Preparation and Sterilisation

Cement discs measuring 7.75 mm diameter by 1.5 mm were cast for each in vitro analysis and allowed to set overnight at 37 °C, in 100% humidity. Discs were disinfected by submergence in 70% ethanol for 30 min, followed by exposure to UV light for 30 min and subsequently rinsed in PBS for 2 min. Discs were then immersed in 1 mL of the cell culture media for 72 h to equilibrate.

##### Cell Culture

Saos-2 cells were cultured in 25 cm^2^ vented cell culture flasks in a humified incubator at 37 °C with 5% CO_2_. Cultures were maintained in minimum essential media alpha medium (Invitrogen Life Technologies, Carlsbad, CA, USA), supplemented with 10% fetal bovine serum (FBS; ThermoFisher Scientific, Auckland, New Zealand) and 1% penicillin−streptomycin antibiotics (Life Technologies, Auckland, New Zealand). Saos-2 cells from passage P4–P8 at 70–80% confluence was seeded directly onto cement disks (6 × 10^3^ cells/disk) for cell viability and proliferation assays. This specific cell density has previously been identified as reaching an appropriate level of confluency over the test period [32,34,35]. Discs were incubated for 1 h to allow the cells to adhere before 1 mL of media was added to cover each disk. Fresh culture medium was replaced daily over the experimental periods (24, 48 or 72 h). Three replicates were used for each BHA cement and MTA, and experiments were conducted 3 times.

##### Cell Proliferation Assay

The cell proliferation of Saos-2 cells grown on the BHA cements and MTA discs was determined using the MTS [3-(4,5-dimethylthiazol-2-yl)-5-(3-caebozymethoxyphenyl)-2-(4-sulphonyl)-2H-tetrazolium] assay at 24, 48 and 72 h. The cell proliferation was assessed by calorimetric measurement of the samples using a spectrophotometer (Synergy 2 Multi-Mode Microplate Reader, Biotek**^®^**, Tokyo, Japan) at a wavelength of 490 nm. Values were compared against the standard curve to calculate cell numbers [41]. 

##### LIVE/DEAD Assay

The cell viability of Saos-2 cells on the BHA cements and MTA discs were evaluated after 48 h cultures using the LIVE/DEAD**^®^** cytotoxicity assay (Invitrogen Life Technologies). Fluorescent visualisation and image capture was performed using an Olympus AX70 (Olympus Corporation, Tokyo, Japan), as previously described [41].

##### Statistical Analyses

Data were evaluated using SPSS version 24 (IBM Corp) and Prism (Graph Pad Prism 6, San Diego, CA, USA) statistical software. Bar graphs were reported as + standard error of the mean. Nonlinear curves were represented by their equations and coefficient of determination (r^2^) values. Statistically significant differences were determined using one-way analysis of variance (ANOVA). If differences were detected, multiple comparisons were made using Tukey’s multiple comparison tests at a confidence level of 95% (*p* < 0.05).

## 3. Results

### 3.1. Chemical Characterisation

#### 3.1.1. ICP-MS Analysis

The acid soluble content of lead (Pb: 3.1 mg/kg) and arsenic (As: 6.0 mg/kg) was determined via ICP-MS (Agilent 7500cs) analysis of the BHA 10% cement after 28 days of immersion in distilled water. Table 2 summarises the elemental composition of the BHA 10% cement. This section may be divided by subheadings. It should provide a concise and precise description of the experimental results, their interpretation, as well as the experimental conclusions that can be drawn.

#### 3.1.2. Fourier Transform Infrared Spectroscopy (FTIR)

**Figure 1:**
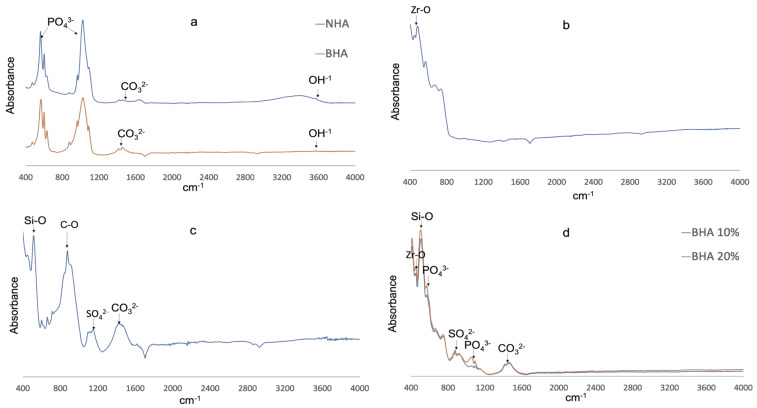
FTIR Spectra. (**a**) NHA (blue) and BHA (red). (**b**) Zirconia dioxide. (**c**) Portland cement. (**d**) BHA10% (blue) and BHA 20% (red).

The FT-IR spectra of NHA, BHA, PC, ZrO_2_ and BHA 10% and 20% cements are presented in Figure 1. NHA and BHA demonstrated prominent bands associated with hydroxyapatite (Phosphate, carbonate and hydroxyl) [32,35]. Portland cement demonstrated peaks at (i) 518 cm**^−^**^1^ with the vibration of Si-O, (ii) 875 cm**^−^**^1^ with the consistent vibration of C–O with CO_3_^2−^, (iii) 1155 cm^−1^ consistent with S–O stretching of SO_4_^2−^, and (iv) 1425 cm**^−^**^1^ consistent with symmetric stretching of CO_3_^2−^ [42]. Zirconia dioxide demonstrated a peak at 483 cm**^−^**^1^ consistent with the stretching vibration of Zr-O of the ZrO_2_ phase [43]. BHA 10% and 20% cements demonstrated peaks overlapping the spectra of BHA, ZrO_2_ and PC forming a composite of the combined elements.

#### 3.1.3. SEM and EDX Analysis

**Figure 2:**
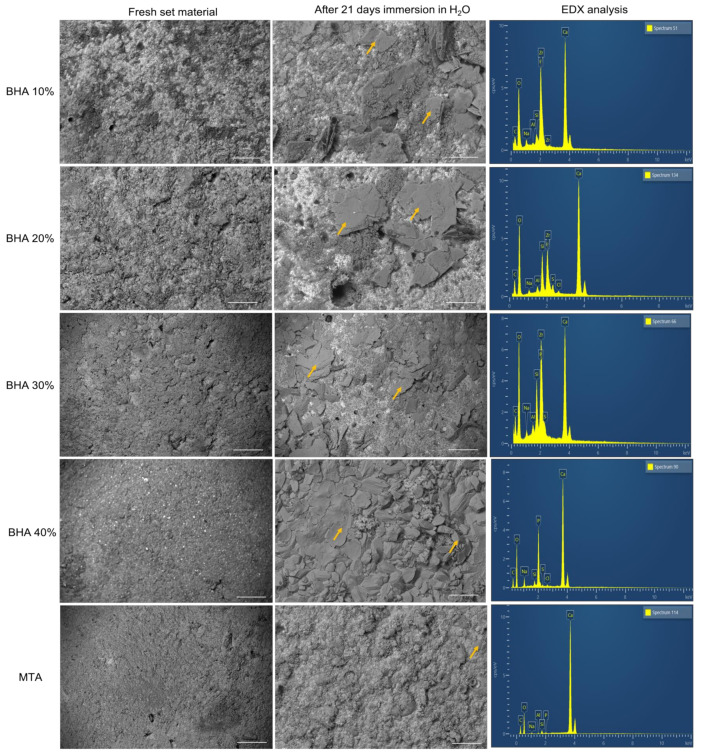
SEM images and their respective EDX analysis of the cements after initial set and 21 days immersion in H_2_O. Yellow arrows indicate apatite formation. (Error bar = 10 µm).

SEM images demonstrate the different surface topographies corresponding with the different cements studied (Figure 2). SEM images of specimens after 21 days of immersion in distilled water, indicated that all groups showed surface deposition (yellow Arrows); which was identified as being apatite particles (calcium phosphate peaks) by EDX analysis (Figure 2).

### 3.2. Mechanical and Physicochemical Properties

#### 3.2.1. Compressive Strength

**Figure 3:**
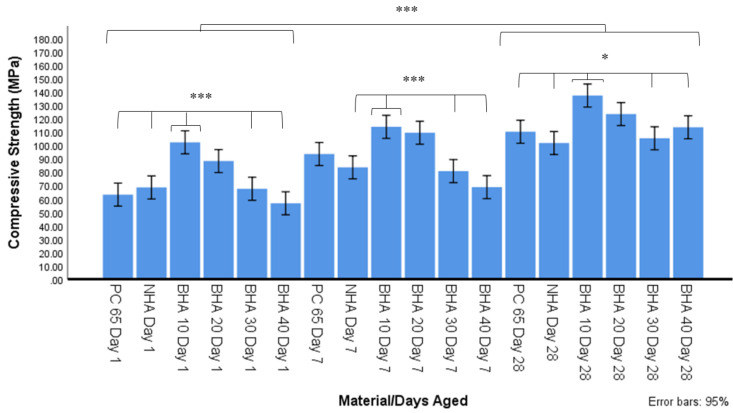
Compressive strength after 1, 7 and 28 days ageing in distilled water using ISO 9917-1 (2007) (n = 10). Significant differences between materials from day 1–28 (*p* < 0.001), on day 1 between BHA 10% and PC 65, NHA, BHA 30% & 40% groups (*** *p* < 0.001), on day 7 between BHA 10% and NHA, BHA 30% and BHA 40% (*** *p* < 0.001), and day 28 between BHA 10 and PC65, NHA, BHA 30% and BHA 40% (* *p* < 0.05).

A significant increase in compressive strength within each experimental group was observed: (i) from days 1–7 in PC (*p* = 0.002) material groups; (ii) between days 1 and 28 in all material groups (*p* < 0.001); and (iii) from days 7 to 28 in BHA 40% (*p* < 0.001) material groups. The BHA 10% material was significantly stronger than NHA, BHA 30%, BHA 40% at all time points studied, and PC 65 at days 1 and 28 (*p* = 0.005) (Figure 3). The BHA 20% material was significantly stronger at day 1 compared with PC and BHA 40% (*p* < 0.01), and at day 7 compared with NHA, BHA 30% and BHA 40% (*p* = 0.005).

#### 3.2.2. Radiopacity

**Figure 4:**
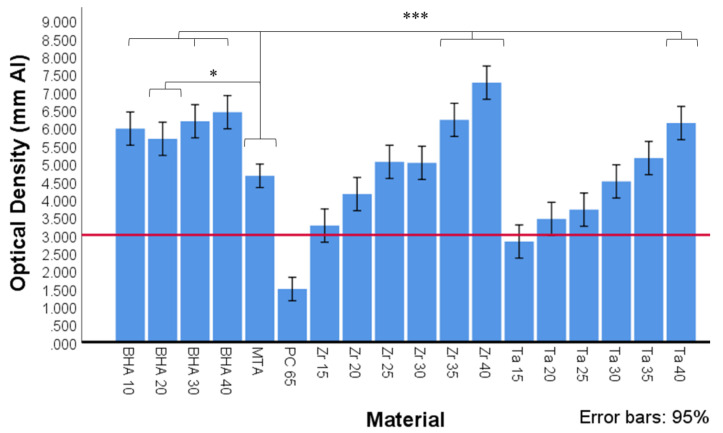
Optical density expressed in equivalent thickness of aluminium (Al) of MTA, Portland cement (PC) and PC with different radiopacifiers, as determined by ImageJ software (n = 3). All BHA groups contained 35% ZrO_2_. Red line depicts ISO 9917-1 (2007) cut off at 3 mm Al equivalent thickness. Significant differences between MTA and BHA 10%, BHA 30% and BHA 40%, ZR 35%, Zr 40% and Ta40 (*** *p* < 0.001) and MTA and BHA 20% (* *p* < 0.05).

The mean optical density values in mm Al equivalent thickness of BHA 10%, BHA 20%, BHA 30%, BHA 40%, MTA and PC radiopacified with ZrO_2_ or Ta_2_O_5_ are presented in Figure 4. There was no significant difference between the different BHA groups in terms of radiopacity (equivalent mm Al thickness). All BHA groups exhibited significantly higher radiopacity than ProRoot MTA (*p* < 0.05). No significant difference in radiopacity was observed between the MTA and the Zr 20%, Zr 25%, Zr 30%, Ta 25%, Ta 30% and Ta 35% containing material groups. MTA exhibited significantly lower radiopacity compared with the Zr 35%, Zr 40% and Ta 40% groups (*p* < 0.001).

#### 3.2.3. Setting Time

**Figure 5:**
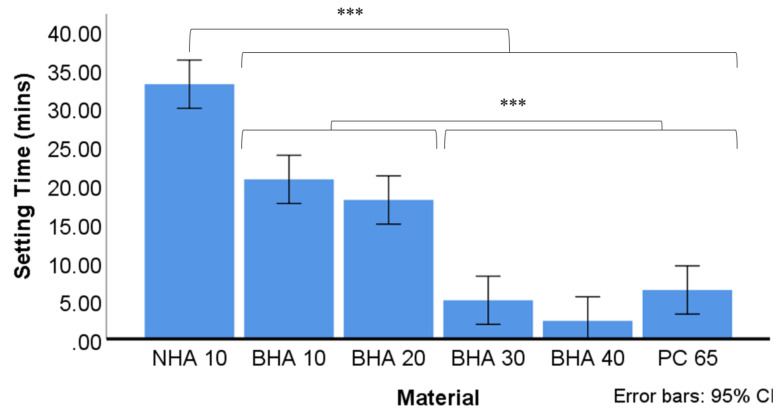
Average setting time of the tested cements (n = 3). Time recorded based on average of three tests using Vicat apparatus. The final time recorded when needle was unable to make complete circular indentation in setting cement. Significant differences between NHA and all other groups *** *p <* 0.001, BHA 10% & BHA 20%, and between BHA 30%, BHA 40% & PC 65. *** *p <* 0.001. * *p <* 0.05, ** *p* < 0.01, *** *p <* 0.001.

The average setting time for each material is summarised in Figure 5. The BHA 40%, BHA 30% and PC65 materials set significantly quicker than all other groups studied. The BHA 20% and BHA 10% materials set over a significantly longer timeframe compared with these groups, although they set more rapidly than NHA which exhibited the longest setting time.

#### 3.2.4. Solubility and pH

**Figure 6:**
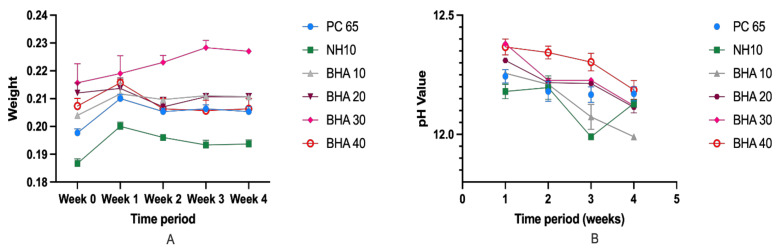
(**A**) Average pH value of the tested cements for the duration of 4 weeks. (**B**) Mean weight loss of the materials studied over the four-week period. (n = 3).

There was no significant change in the mean weight of the materials studied over the four-week period (Figure 6). Furthermore, there were no significant differences in the pH values within the groups over the four-week period. However, except for BHA 10% (pH 11.99) at week four and NHA (pH 11.96) at week three, all groups maintained an average pH above 12 over the entire test period. The mean pH of NHA (11.96) at week three was significantly lower than the mean pH of BHA 30% at week one (12.38, *p* < 0.024), and BHA 40% at week one (12.39, *p* < 0.034).

### 3.3. In Vitro Biocompatibility Testing

#### Cell Proliferation Assay and LIVE/DEAD Assay

The cell proliferation and biocompatibility of the BHA cements and MTA were investigated using the MTS assay. The cell numbers increased with time and both the BHA cements and MTA materials were, therefore, considered to be biocompatible for the Saos-2 cells (Figure 6A). Notably the 10% and 20% BHA cements presented higher cell numbers across all three time points compared with MTA, which acted as the control. According to Figure 6A, there was no significant difference observed in cell numbers between the BHA cements and MTA at 24 h and 48 h (ANOVA; *p* = ≤ 0.05). However, there was a significant difference between 10% BHA and MTA (ANOVA; *p* = 0.01), and 20% BHA and MTA at 72 h (ANOVA; *p* = <0.0001). 

**Figure 7:**
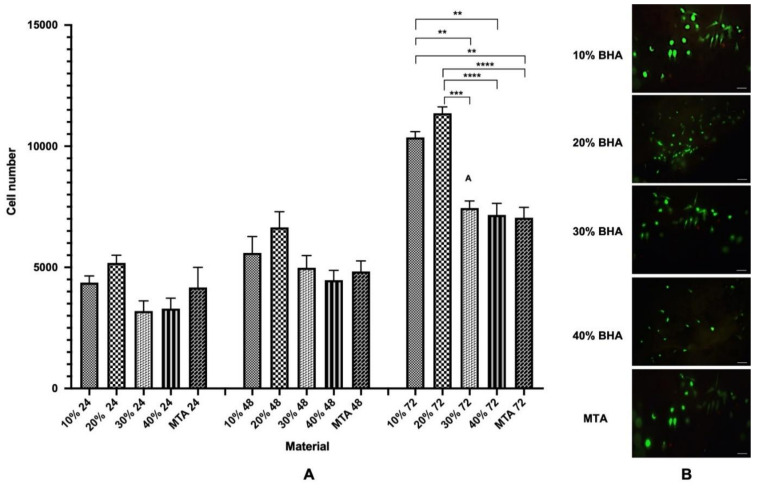
(**A**) Proliferation (MTS assay) of Saos-2 cells after seeding onto BHA cements and MTA after 24, 48 and 72 h. Error bars represent ± SE of the mean after 1-way ANOVA with Tukey’s multiple comparison test (n = 3, ** *p* < 0.01, *** *p* < 0.001, **** *p* < 0.0001 and error bars represent the SE of the mean). (**B**) Fluorescent images showing cell viability of Saos-2 cells seeded on the BHA cements and MTA discs after 48 h (n = 3). Green = live cells (calcein), Red = dead cells (ethidium homodimer-1). Scale bar = 100 μm.

Figure 7B shows the BHA cements and MTA discs seeded with Saos-2 cells after performing the LIVE/DEAD assay after 48 h. Results indicated excellent cell viability for the BHA cements and MTA and both the materials were found to be non-toxic, i.e., few dead cells were apparent. The cells adhered to the surface of both the materials and exhibited a characteristic spindle-like morphology.

## 4. Discussion

Modifications to the composition of calcium silicate endodontic cements affect their biological, chemical and physical properties. Clinically desirable properties of endodontic cements include biocompatibility, antibacterial effects, dimensional stability, radiopacity, and compressive strength. Setting time data presented here indicate that the BHA addition to cements affected the compressive strength, setting time and cell growth, but did not significantly alter radiopacity, pH or solubility. 

ICP-MS analysis showed that the BHA 10% cement consists mainly of calcium and phosphate particles with trace amounts of sodium and magnesium, which is found with bovine derived hydroxyapatite [32,35]. Analysis of the ground cement powder revealed the acid leachable arsenic content was higher than the allowed levels under ISO 9917-1 guidelines [36] (Table 2). The relevance of the ISO 9917-1 method has been questioned, as crushing cement samples greatly increases the surface area beyond what would normally occur clinically [44]. Primus suggests even if all arsenic present in samples was released, it would still be below the maximum safe daily exposure level recommended by the World Health Organisation [44]. Further research is required to establish whether the allowed maximum arsenic levels have any relevance as a risk to human health when used clinically.

FTIR spectra of the BHA and NHA exhibited a similar spectrum showing the characteristic peaks of HA, and indicating the processing of bovine bone was successful in removing fat and protein components (Figure 1a). A pronounced peak was observed at 3570 cm^−1^ due to the presence of the hydroxyl group. Phosphate bands were observed at 1092 to 1040 cm^−1^, 962 cm^−1^, 633 to 566 cm^−1^ and 473 cm^−1^, respectively. Carbonate was retained within the BHA structure which was confirmed by the bands between the region at 1455–1418 cm^−1^ and 873 cm^−1^. A similar FTIR spectrum was also observed in our previous studies [32,35]. Furthermore, FTIR analysis of PC and ZrO_2_, confirmed the samples corresponded with FT-IR spectra observed in reference materials from previous studies [42,43]. The FTIR spectra for BHA 10% and 20% cements demonstrated peaks associated with BHA (Phosphate, carbonate), ZrO_2_ (stretching vibration of Zr–O of ZrO_2_) and PC (Si–O, C–O and S–O stretching) forming a composite of the combined elements (Figure 1d). 

The test cements and MTA all demonstrated nucleation of apatite on the materials’ surfaces after 21 days of immersion in distilled water, which was confirmed by EDX analysis (Figure 2). 

An increase in compressive strength was observed in all cements tested between days 1 and 28. This was expected due to the continuation of cement hydration and curing [45]. BHA 10% exhibited the most consistent improvement in strength, outperforming NHA, BHA 30% and BHA 40% at all time points, and PC65 at day 1 and 28. BHA 20% also performed well in compressive strength tests and was significantly stronger than multiple cements at the day 1 (PC and BHA 40%) and 7 (NHA, BHA 30% and BHA 40%) time points. The addition of 10% NHA did not improve compressive strength significantly, however, it did not significantly reduce compressive strength either, as has been observed by Guerriro-Tanomaru and Vazquez-Garcia [31]. Improved compressive strength of BHA cements with 10% and 20% concentrations, and a reduction in strength in concentrations above 30% is in agreement with the findings of Lu and Zhou [20]. The mechanism by which BHA was able to alter the compressive strength of these materials is unknown. One possibility for this outcome is that the particles act as a nucleating agent to facilitate hydration of other cement particles. In Biodentine, calcium carbonate is added as it does not react with water and acts as a nucleation site to enhance cement hydration, resulting in a material with increased density and compressive strength [46]. Amorphous BHA, similar to calcium carbonate, is stable in an aqueous solution and has no cementitious qualities. At lower concentrations of 10% and 20% BHA may improve cement hydration through this mechanism. At concentrations above 20% BHA a reduction in the amount of reinforcing cement matrix formed may occur which could reduce overall cement strength. 

MTA was originally developed for use as a root end filling material due to its excellent sealing properties and ability to set in a moist environment [47]. These same properties also contribute to the success of MTA as a perforation repair material and pulp capping agent [47,48]. Compressive strength of MTA is a more important material attribute when used in coronal regions than apically [49]. Consequently, BHA 10% and 20% cements would, therefore, be potentially better suited as base materials and pulp capping agents than BHA 30% and 40% cements. 

The addition of different concentrations and types of hydroxyapatite did not significantly alter the radiopacity of the cements. Tantalum pentoxide and zirconium dioxide are commonly used as non-discolouring radiopacifiers in CSCs; however, the proportion added varies with different manufacturers. Using readily available materials, this study determined that the addition of concentrations above 20% for ZrO_2_ and 30% for Ta_2_O_5_ resulted in a radiographic density equivalent to that of ProRoot MTA. These results suggest ZrO_2_ may provide a better option than Ta_2_O_5_ as a smaller quantity is required to achieve ideal radiopacity, allowing for a higher % wt content of cementitious materials. 

The addition of hydroxyapatite at lower concentrations delayed the setting time of the material. However, when incorporated at higher concentrations the setting time was reduced. Consistent with other studies the use of a plasticising agent reduced water requirements and improved material handling [50]. Calcium phosphates (i.e., CPM), however, can increase the water demands of CSCs and alter the rheological properties of cements [51]. The amount of water required to achieve a putty-like consistency, however, differed between groups. NHA had the highest water demands and longest setting time, followed by the BHA 10%, BHA 20%, BHA 30% and 40% groups, respectively. Notably, the setting time of MTA has been shown to increase with a higher water content [52]. The smaller particle size of NHA additives may have altered the cements rheological properties and relative water requirements, resulting in increased water demands and setting time. In certain clinical applications, such as endodontic surgery, the rapid setting properties of BHA 30% and BHA 40% setting cement can be advantageous in reducing material washout and dissolution [49].

In vital pulp therapy (VPT), the use of a dental dam for isolation, reduces the potential for material washout during placement. Slow setting time appears to be less important when calcium silicates are used in VPT. Tsujimoto et al. demonstrated that teeth treated with MTA for pulp capping could be restored immediately with bonded composite resin restorations without negatively affecting the material’s hardness [53]. The longer setting time of BHA 10% and BHA 20% cements, therefore, may be acceptable when used for VPT or the repair of furcal perforations as in these areas of the tooth, the compressive strength is more important than a rapid setting time.

The minor changes in pH and material weight observed over four weeks indicated that the materials were dimensionally stable. Indeed, BHA incorporation had minimal, if any, effect on the material’s pH over time. A pH of >12 was recorded at every time point for all but three groups (see Section 3.2.4, although the pH value was relatively close to pH 12. Clinically, these finding indicate that BHA addition should not reduce the antimicrobial properties associated with the material’s high setting pH [12]. However, a limitation of this study is that the antimicrobial activity was not investigated which should be a future direction. 

The viability of the cells on the BHA cements were assessed using a LIVE/DEAD cytotoxicity assay kit. The BHA cements were non-toxic, allowing the cells to adhere to and support the growth of Saos-2 cells. Indeed, dead cells were present only in low numbers (Figure 7B). The ability of the BHA cements to promote cell growth was assessed using the MTS assay (Figure 7A). Cell numbers increased with culture time for both the materials, suggesting that the scaffolds enhanced cells adherence and facilitated cell proliferation. Although there was no statistically significant difference between the BHA cements and MTA at 24 h and 48 h, interestingly, both 10% BHA and 20% BHA exhibited a higher cell density compared with MTA. Remarkably, 10% BHA and 20% BHA exhibited a significantly higher cell density compared with MTA at 72 h. Notably, the data indicates that the addition of BHA had a stimulatory effect on cell growth, potentially providing a substratum for osteoconduction. Furthermore, trace elements, such as carbonate, sodium, and magnesium, found in BHA influence various biomechanical reactions linked with cell proliferation and differentiation [32,35].

## 5. Conclusions

The study demonstrated the development of cheaper and superior endodontic materials than MTA for endodontic applications using waste bovine bone. The compressive strength of experimental cements was increased with the addition of 10–20% BHA, without affecting radiopacity, solubility or pH. However, in the BHA 10% and 20% supplemented groups, the setting time was significantly longer than in the BHA 30% and 40% materials. Furthermore, at BHA concentrations of 10% and 20%, cell growth of Saos-2 cells was significantly improved compared with MTA, and 30% and 40% BHA groups after 72 h incubation. These results indicate that biologically sourced HA provides a viable alternative to CPM as an additive to CSCs. However, further in-vivo studies are necessary to investigate the BHA cements’ clinical feasibility as pulp capping or vital pulp therapy agents.

## Figures and Tables

**Table 1:** Cement powder ratios/liquid ratios per group.

	1 g Powder	Liquid (g)
PC65	0% HA: 65% PC: 35% Zr	0.15
NHA	10% NHA: 55%PC: 35% Zr	0.24
BHA10%	10% BHA: 55%PC: 35% Zr	0.15
BHA20%	20% BHA: 45%PC: 35% Zr	0.15
BHA30%	30% BHA: 35%PC: 35% Zr	0.14
BHA40%	40% BHA: 25% PC: 35% Zr	0.14

**Table 2:** Acid soluble content of BHA 10% cement determined by ICP-MS after 28 days immersion in H_2_0.

Element (mg/kg)	BHA 10%	Maximum Limit (ISO 99107-1 2007)
Pb	3.1	100
Ca	260,000	
P	14,000	
Al	8700	
Fe	8700	
Mg	3100	
Na	1100	
Ba	230	
Zn	49	
Cr	18	
Ni	10	
Cd	0.2	
Hg	<0.006	

## Data Availability

The data presented in this study are available on request from the corresponding author.
